# A generalized deceptive pollination system of *Doritis pulcherrima *(Aeridinae: Orchidaceae) with non-reconfigured pollinaria

**DOI:** 10.1186/1471-2229-12-67

**Published:** 2012-07-03

**Authors:** Jin Xiaohua, Li Dezhu, Ren Zongxin, Xiang Xiaoguo

**Affiliations:** 1 State Key Laboratory of Systematic and Evolutionary Botany & Herbarium, Institute of Botany, Chinese Academy of Sciences, Beijing, 100093, China; 2 Plant Germplasm and Genomics Center, Germplasm Bank of Wild Species, Kunming Institute of Botany, Chinese Academy of Sciences, Kunming, Yunnan, 650201, China

**Keywords:** Aeridinae, *Doritis pulcherrima*, floral deception, Orchidaceae, pollinarium reconfiguration

## Abstract

**Background:**

As one of largest angiosperm families, orchids have long fascinated evolutionary biologists with their staggering diversity in floral design and display to promote outcrossing. Two of the most intriguing aspects of orchid pollination that promote cross-pollination are pollinarium reconfiguration (PR) and deceptive pollination. PR and generalized food deception employ virtually antagonistic methods of promoting cross-pollination: PR occurs through delayed pollination, involving the relatively long visitation periods that are typically observed for the pollinators of one flower or inflorescence; conversely, generalized food deception leads to reductions in the visitation periods of pollinators to one flower or inflorescence. Thus, it is logical to hypothesize that PR is unnecessary or PR happens soon in generalized food-deceptive orchids in the promotion of cross-pollination. Using *Doritis pulcherrima* as a model, the aim of this study was to understand the following: (1) the pollination and breeding system of *D. pulcherrima*; (2) the morphological interactions between orchids and their pollinators; and (3) whether PR is necessary in the promotion of cross-pollination in *D. pulcherrima*.

**Results:**

Our observations indicated that *Doritis pulcherrima* is pollinated almost exclusively by *Amegilla nigritar* (Hymenoptera: Apidae) and possesses pollinia that are deposited on the “occiputs” (cervical membranes) of these insects. All of evidences are indicated that *D. pulcherrima* is a generalized food-deceptive orchid. Our morphometric measurements of the flowers and pollinators show that the heights of the “occiputs” with un-oriented pollinaria were equal to the distances between stigmas and surfaces of the middle lobes, suggesting that pollinarium reconfiguration is not necessary in *Doritis pulcherrima*.

**Conclusions:**

Our observation and analyses supported the hypothesis that pollinarium reconfiguration is unnecessary in generalized food-deceptive orchids, such as *Doritis pulcherrima*, for the promotion of cross-pollination. This conclusion was indirectly supported by the abundance of deceptive orchids that do not exhibit pollinarium reconfiguration. There are two mechanisms (i.e. clone-growing characteristics and a long flowering season) that promote fruit sets in the epiphytic food-deceptive orchids in tropical regions.

## Background

As one of largest plant families with approximately 19,500 species [1], orchids have long fascinated evolutionary biologists with their staggering diversity in floral design and display to maximize outcrossing. With the exception of a few autogamous species, most orchids require pollinators for fruit set, and two of the most intriguing aspects of orchid pollination that promote cross-pollination are pollinarium reconfiguration (PR) and deceptive pollination. PR refers to the change in orientation that a pollinarium undergoes following its removal and prior to its deposition in the stigma area [2,3]. The term PR includes pollinarium bending (PB), pollinium shrinking (PS) and anther cap retention (ACR). PB, also referred to as re-orientation, is the most widespread form of PR and was first described by Darwin [4]. PB is hypothesized to prevent pollinator-mediated self-pollination by retarding pollen deposition until the pollinators have moved to another plant. Peter and Johnson [3] provided evidence in support of Darwin’s hypothesis. PS was reported in two species of *Bulbophyllum*[5] and *Trigonidium*[6], whereas ACR has been recorded in several genera, including *Eulophia*[3] and *Tipularia*[7].

It is estimated that approximately one-third of orchids, approximately 6500 species, are pollinated through deception [8-12]. Jersáková et al. [13] listed seven deception mechanisms in orchids; of these generalized food deception is the most common, occurring in 90 % of all deceptive orchids. In contrast to PR, deceptive pollination promotes cross-pollination by reducing the visitation period of pollinators and/or discouraging repeat visits to one flower or one inflorescence [9,14,15].

PR and generalized food deception employ virtually antagonistic strategies of promoting cross-pollination: PR occurs through delayed pollination, involving the relatively long visitation periods that are typically observed in the pollinators of one flower or inflorescence; conversely, generalized food deception leads to reductions in the visiting periods of pollinators to one flower or inflorescence even a patch of plants. Thus, it is logical to hypothesize that PR is unnecessary or PR happens soon in generalized food-deceptive orchids in the promotion of cross-pollination. It is, thus, interesting to note that several large genera in Orchidaceae, such as *Bulbophyllum* (approximately 1500–2000 species), *Dendrobium* (approximately 800–1200 species), and *Eria* s.l. (approximately 400–500 species), are characterized by their rewardless flowers and naked or nearly naked pollinaria lacking stipes for which PR cannot occur (*Bulbophyllum*[13,16]; *Dendrobium*[17,18]; *Eria*[13,19]). However, there are very few studies focusing on the causal association among the loss of a stipe, deceptive pollination, and the PR.

The evolutionary radiation of the subtribe Aeridinae in tropical Asia is characterized by floral divergence and adaptation to pollinators, especially with respect to the hard pollinium and rather long stipe [20-25]. Our fieldwork observations and literature investigations indicated that pollinaria do not undergo reconfiguration (or at least PB or PS is not employed) in some of the genera of Aeridinae, including *Acampe* (approximately 10 species)*, Cleisostoma* (approximately 100 species)*, Doritis* (1 species)*, Hygrochilus*(1 species), *Papilioanthe* (approximately 10 species)*, Renanthera* (approximately 10 species)*, Staurochilus* (approximately 10 species)*, Thrixspermum* (approximately 120 species)*,* and *Vandopsis* (approximately 10 species). In addition, most of the species in these genera are rewardless [20-28], but see [29].

Using *Doritis pulcherrima* as a model, the aims of this study are to understand the following: (1) the pollination and breeding system of *D. pulcherrima*; (2) the morphological interactions between orchids and their pollinators; and (3) whether PR is necessary in the promotion of cross-pollination in *D. pulcherrima*.

## Materials and methods

### Materials

**Figure 1:**
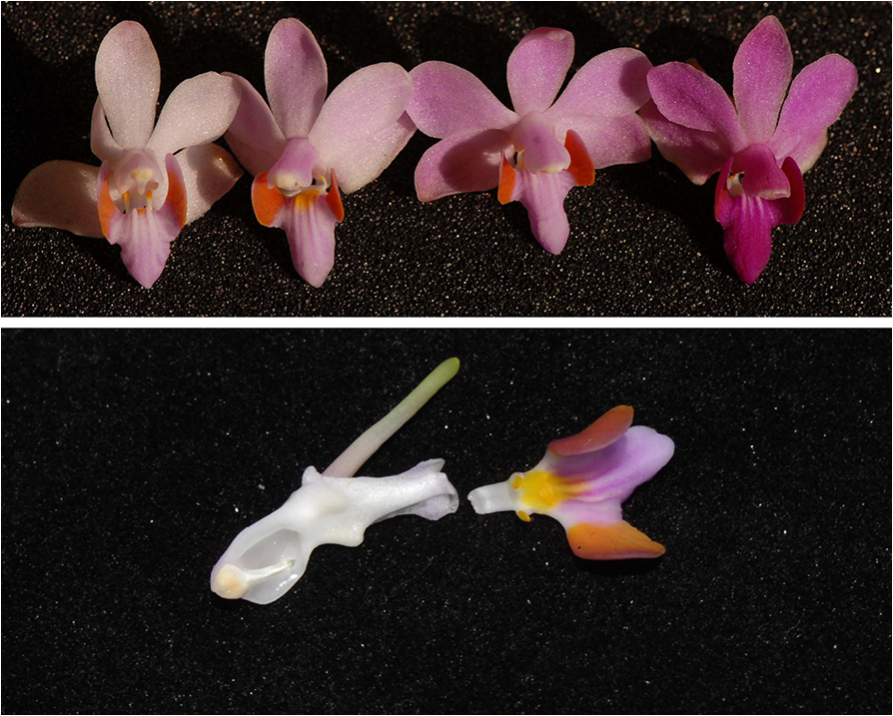
**The variation of flower color and pseudopollen in*Doritis pulcherrima.***

*Doritis pulcherrima* Lindl. (*Phalaenopsis pulcherrima* (Lindl.) J.J. Sm., voucher specimens, Hong Kong Kadoorie Program Team 5355, deposited in PE) is widespread in tropical Asia, including India, Myanmar, Vietnam, Laos, Thailand, Malaysia and China [20,24]. The species mainly forms clones on marble outcrops at the borders between rocks and soil, among the bushes or under sparse forests at elevations of 200 to 500 m in the lowland tropical monsoon rainforests of the Hainan Island, China. Each stem produces 1–2 young shoots every year, forming dense clones with many shoots; the flowering period occurs from July to October. The flower colors vary from white and pale pink to darker pink, red and purple (Figure 1). The lip attaches to the end of the column foot and is clawed and tri-lobed, containing pairs of pollen-like yellow calli on the claw and yellow appendages with blots in front of them (Figure 1). The lateral lobes are erect, while the middle lobes are deflexed. Four yellowish pollinia are situated on a long and linear stipe. The pollinaria of *Doritis pulcherrima* are long. Our previous field investigation indicated that they do not undergo reconfiguration when they are removed from the columns. No nectar has been reported in literatures to date [20,21,24].

### Study sites

This study was conducted using three populations from the southern and southwestern region of Hainan, China: the Yang-lin population (S1, elevation 386 m, 18°30’ N, 109°13’ E, approximately 2000 clones); the Wang-xia population (S2, elevation 469 m, 19°01’ N, 109°07’ E, approximately 320 clones); and the Ma-an-shi population (S3, elevation 200 m, 18°44’ N, 108°59’ E, approximately 140 clones). The climates of these three localities are tropical and strongly seasonal (for S1, the mean annual temperature is 25.8°C and the mean annual precipitation is 1392 mm and concentrated between June and October).

Pollinator observations were conducted in these three populations to reveal the pollinator specificities. The sizes of each of the *D. pulcherrima* clones were evaluated for S2, whereas the breeding system experiments were conducted using S1.

### Visitor observations

The behaviors of the flower visitors were observed during the day and night for the three populations between June and September of 2004 and July and August of 2005. The diurnal observation time was from 6:00 am to 20:00 pm (186 h in total), and the remaining observations were nocturnal (61 h in total). We recorded the behavior of the pollinators on *D. pulcherrima*, including the visitation behaviors, number of inflorescences and flowers visited per cluster, numbers of flowers visited per inflorescence, visitation period on each flower and inflorescence and numbers of pollinaria on each pollinator, were recorded. The visitors were collected from *D. pulcherrima* inflorescences and other co-blooming plants using nets and were killed with ether using a killing-bottle. The insects were identified by entomologists of the Entomology Department of Southwestern Forest University, Kunming, Yunnan, China. The insect voucher specimens (Jin X.H. P4, P7, P9, P15, and P20) were deposited in the Herbarium (PE) at the Institute of Botany, Chinese Academy of Sciences.

### Measurements of the functional characteristics of flowers and pollinators

Ten full-blooming flowers were randomly selected from the Yang-lin population in 2010, and their functional characteristics were measured. The entrance and floral passage that were formed by the two lateral lobes of the lips (sides), columns (top) and lip claws (bottom) were considered to be the key functional characteristics of *D. pulcherrima* that would aid in the understanding of the morphological interactions between the plant and its pollinators. Four functional characteristics that were related to pollinia transfer and receipt were measured: the height (distance between the rostellum and the surface of the middle lobe), width (distance between the two lateral lobes of the lip at the entrance of the passage), depth (distance between the entrance and lip claw), and the distance between the stigma and surface of the lip. Six characteristics closely related to pollen transfer were measured: the width and height of the head, the height and width of the thorax, the length of the proboscis, and the total height of the pollinarium and position of attachment of the pollinarium to the pollinator. All of the above variables were measured to an accuracy of 0.1 mm using dial calipers (Zhengjiang Qiaosheng, HU 02270108).

### Pollinarium reconfiguration

To test whether the pollinaria of *D. pulcherrima* show PR, time-lapse photography (Nikon D700 digital camera on a tripod) was used in the natural habitat of *Doritis pulcherrima* (the S2 population ) during September of 2011. For ACR, ten pollinaria were carefully removed with toothpicks, and then photographs were taken every 5 seconds for 10 minutes of the anther caps that contained pollinaria. To observe PB and PS, photographs were taken every 5–10 seconds for 20 minutes of ten pollinaria that were positioned with toothpicks and had their anther caps removed with forceps.

### Floral biology

Due to the densely branched, growing habitat of *D. pulcherrima*, we decided to use each clone as a minimum unit in our phenology census for the sake not to disturb the clones during the investigation. For the phenology census and natural fruit set assessments, 266 flowers from 21 clones in 2004 and 638 flowers from 44 clones in 2005 were randomly selected and marked as S1. Because the flowering season of *D. pulcherrima* extends into the monsoon season of Asia, only the following variables were recorded: (a) the numbers of flowers on each inflorescence and clone, which were checked once at the beginning of the investigation and counted as fertile bracts or flowers buds; (b) the numbers of open flowers on each inflorescence and clone, which were measured twice a week; and (c) the numbers of fruits (immature fruit) on each clone, which were obtained at the end of October in 2004 and August in 2005.

### Floral rewards

The floral rewards of *Doritis pulcherrima* were examined using S2 population in 2011. To measure the nectar concentrations and volumes, we randomly selected ten flowers and bagged them with nylon net bags before they opened. After anthesis, the flowers were removed to measure the nectar secretions. The presence/absence of nectar was examined visually and using an optical microscope and the 5 μL SIGMA micro-cap calibrated capillary tubes (Sigma-Aldrich, St. Louis, MI, USA). The volumes were determined by measuring the lengths of the filled tubes and converting the length measurements to microliters. To examine whether the pseudopollen on the lips was collected by pollinators during their visits, twenty S1 flowers whose pollinia had been removed by pollinators were examined using an optical microscope in 2005.

The presence/absence of nectar of the five co-blooming species, including two species of *Lasianthus* (Rubiaceae), one species of *Leptodermis* (Rubiaceae), one species *Polygala* (Polygalaceae), and one species of *Helicteres* (Sterculiaceae), visited by the pollinators were examined using an optical microscope in 2011.

### Breeding system

The breeding system of *Doritis pulcherrima* was evaluated by controlled bagging in 2005 for S1. The treatments included artificial self-pollination (pollinia from the same clone), cross-pollination, and unmanipulated flowers (control). Pollinaria for the cross-pollination were obtained from plants that were situated over 20 m away. The numbers of clones, inflorescences and flowers that were used in each treatment are summarized in Table 1.

**Table 1:** Percentage of fruiting success per treatment and natural fruit set in S1

	Number of cluster	Number of flowers	Fruit set [%]
Bagged	11	22	0
Hand self-pollination	11	26	42.31
Hand cross-pollination	18	23	73.91
Natural pollination in 2004	21	266	13.16
Natural pollination in 2005	38	578	10.98

### Statistic analyses

All of the analyses were conducted using SPSS 16.0 for Windows. The data were analyzed using descriptive statistics. The variations in the natural fruit sets over the 2 years were analyzed using a one-way analysis of variance (ANOVA).

## Results

### Phenology

Each shoot produces 1–2 inflorescences, each of which bore 2–21 flowers (mean ± SD, 8.1 ± 3.9, n = 75), and each clone contains 1–8 inflorescences (mean ± SD, 2.5 ± 1.7, n = 44). The flowers open and age sequentially from their bases; however, only one to three flowers bloomed synchronously on each inflorescence. The non-pollinated flowers last for approximately 10 days and then withered and dropped from the rachises along with their ovaries and pedicels. The life-spans of the cross-pollinated flowers were brief; the cross-pollinated flowers, withering within 2–3 days, whereas the perianth persisted on the young fruit. At the same time, the column wings enlarged to cover the stigmatal areas in one day.

### Visitor behaviors

All of the recorded visitors were diurnal insects, including four species of bees, two species of butterflies, one species of moth and two other unidentified insects. During our observations, 43 of the 77 total recorded visits were by *Amegilla nigritar* Cockerell. in all three of the populations during our observations. The visits are concentrated from 6:30 a.m. to 11:00 a.m.

Among all of the visitors observed, only the bees *Amegilla nigritar* and *Ceratina flavipes* carried pollinaria. However, only *Amegilla nigritar* dislodged the pollinia from the flowers and delivered the pollinia to stigmas, acting as a legitimate pollinator. Although *Ceratina flavipes* carried pollinaria, only one visitation was recorded in S3 in August of 2004, which indicated that this was a rare occurrence.

### Floral rewards

No nectar was detected visually or using an optical microscope, and none was obtained using capillary tubes in the ten examined orchid flowers. Additionally, the pseudopollen was intact with no signs of having been collected. In contrast, nectar has been detected in all five of the co-blooming species visited by the pollinators.

### Pollinarium reconfiguration

The results of the time-lapse photography indicated that ten of the observed pollinaria remained in the same position after 20 minutes, suggesting that they did not undergo shrinkage or reconfiguration. The anther caps of nine of the tested pollinaria remained on the columns when the pollinaria were removed, and only one detaching with pollinarium and then dropped from the pollinarium 2 seconds later.

### Visiting behavior of *Amegilla nigritar*

**Figure 2:**
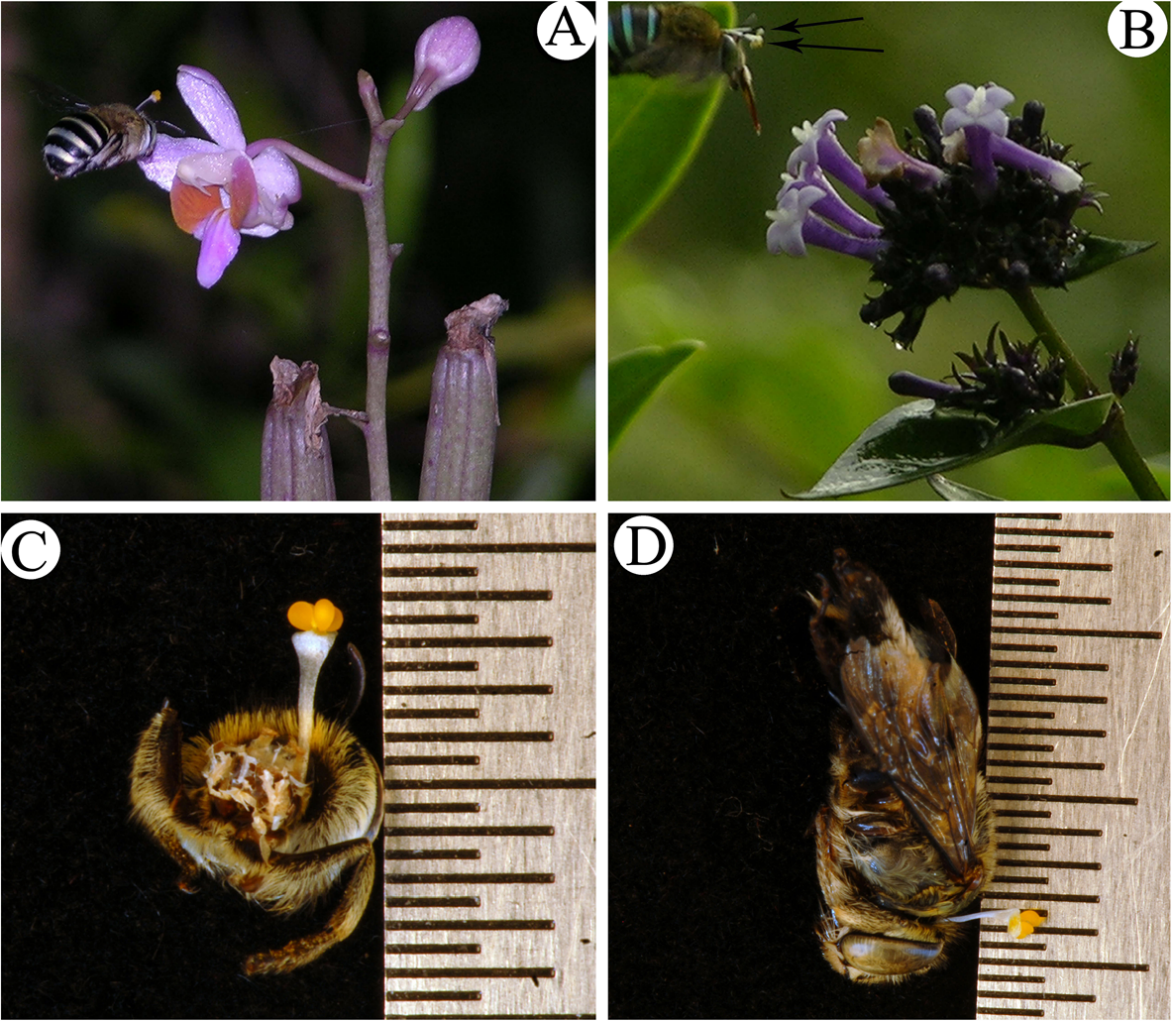
**The visiting behaviors of*Amegilla nigritar*and their positioning of the pollinaria. (A)***Amegilla nigritar* bearing a pollinarium on its neck while visiting a *Doritis pulcherrima* flower. **(B)***Amegilla nigritar* visiting a rewarding plant (*Leptodermis* sp.) with arrows showing stipes. **(C)** Posterior view of *Amegilla nigritar* head showing the attachment of a pollinarium to its neck. **(D)** Lateral view of *Amegilla nigritar* bearing a pollinarium on its occiput.

The pollinators flew low in the forests and patrolled among the bushes and trees. Our observation indicated that about approximately one-sixth of the pollinators visited the flowers of the *D. pulcherrima* inflorescences. Their visits to *D. pulcherrima* were very common foraging manner: the bees landed on the midlobes and then entered the passages; and 1–2 seconds later, the bees retreated from the passages to the midlobes and flew away. The visitations lasted approximately 2–3 seconds, and approximately one-third of the bee’s body entered the passages. During the pollinators’ retreats from the passages to the midlobes, the viscidia attached to pollinator’s occiputs and were removed when they moved on to the next flower. When a pollinator with a pollinarium on its occiput visited another *Doritis pulcherrima* flower, it was able to pollinate it (Figure 2). During our field observations, no pollinators were observed to visit two or more flowers from of same clone.

*Amegilla* bees were observed visiting *D. pulcherrima* as well as the flowers of Rubiaceae (Figure 2B.), Gesneriaceae, Fabaceae, Sterculiaceae, Commeliaceae and other nearby plants, although these flowers had completely different shapes, sizes and colors. The colors of these plants ranged from white to red, pink, and purple. Our field observations showed that the putative pseudopollen on the labella of *D. pulcherrima* were not collected or eaten by the pollinators or visitors.

### Morphological traits of flowers and pollinators

The heights and widths of the entrances of the *D. pulcherrima* flowers were 3.5 ± 0.06 mm and 7.3 ± 0.48 mm (n = 10), respectively. The lengths of the entrances and the distances between the stigmatal areas and surfaces of the middle lobes were 7.6 ± 0.36 and 6.1 ± 0.21 mm (n = 10), respectively. The length of the entire pollinarium was 3.95 ± 0.095 mm (n = 8).

The heights of the thoraces and pollinator widths were 4.7 ± 0.15 and 5.2 ± 0.12 mm (n = 4), respectively. The head widths and heights were 4.9 ± 0.58 mm and 3.6 ± 0.058 mm (n = 4), respectively. The proboscis lengths were 4.1 ± 0.1 mm (n = 4). The total heights of the occiputs with pollinaria were 6.1-6.2 mm (n = 2, Figure 2). All of these measurements are presented in Tables 2 and 3.

**Table 2:** **Key morphological traits of*Doritis pulcherrima*(mm, mean ± SD)**

Floral morphology	*D. pulcherrima*(n = 10)
Height of entrance	3.5 ± 0.06
Width of entrance	7.3 ± 0.48
Length of entrance	7.6 ± 0.36
Distance between stigma and margin of middlelobe	6.1 ± 0.21
Length of pollinarium	3.95 ± 0.095

**Table 3:** Key morphological traits of pollinator (mm, mean ± SD)

Pollinator morphology	*Amegilla* (n = 4)
Thorax height	4.7 ± 0.15
Head width	4.9 ± 0.58
Thorax width	5.2 ± 0.12
Proboscis length	4.1 ± 0.1
Head height	3.6 ± 0.058
Total length of the occiput and pollinarium	6.1-6.2

### Pollination success

The natural fruit set was 13.1 % in 2004 (mean ± SD, 13.1 % ± 10.11, n = 21) and 10.9 % in 2005 (mean ± SD, 10.98 % ± 10.9, n = 38, six clusters, a total 60 flowers, of 44 clusters were destroyed during the typhoon season in 2005) (Table 1). There was no significant difference between natural fruit set in 2004 and 2005 (*F* = 0.179, *P* = 0.674). The fruit sets under the self-pollination and cross-pollination treatments were 42.31% and 73.91% (Table 1); respectively. None of the bagged, unmanipulated flowers (controls) set fruit (Table 1).

## Discussion

### Pollination system of *D. pulcherrima*

There is much evidence suggesting that *D. pulcherrima* is a generalized floral-deceptive orchid. First, the nectar examination and the behaviors of pollinators on the orchids indicated that the orchid is rewardless. Second, the pollinators did not collected pseudopollen during their visits. In addition,the behavior of the pollinators indirectly indicated that *D. pulcherrima* exploited the interrelationships between the pollinators and other co-blooming rewarding species, such as *Leptodermis* spp., *Lasianthus* spp. (Rubiaceae), and *Polygala* spp. (Polygalaceae), although this relationship remains to be tested.

Morphological interactions between orchids and their pollinators are crucial to the success of pollination. Pollinarium reconfiguration is considered to be important in the prevention of self-pollination and the success of cross-pollination. Thus, it is interesting to note that the heights of the occiputs of the pollinators with unoriented pollinaria (6.1-6.2 mm) are equal to the distances between the stigma and the surfaces of the middle lobes (Tables 2 and 3), suggesting that an un-oriented pollinarium is a precondition for the success of pollination.

The precise site of pollinarium placement on the pollinator is determined by several factors, such as the body morphology of the pollinator, precision of the pollinia deposition on the stigma area, and visitation behavior. The dorsal surface of the pollinator, including the thorax, eyes, and frons, is more frequently used for pollinarium attachment [16]. Thus far, the positioning of the pollinaria on the occiputs of pollinators is reported for very few orchids [2,30].

It has been recently suggested that *Amegilla* may be the most important pollinator in the forest understory [31-33], particularly in the Oriental (Indomalayan) region [31]. In Africa, several species of *Amegilla* are among the pollinators of some terrestrial orchids and carry pollinaria on their legs or proboscis [34-36]. Although there are very few reports in the literature that describe *Amegilla* as orchid pollinators, our observations indicate that *Doritis pulcherrima* is exclusively or mostly pollinated by *Amegilla* in Hainan, suggesting that *Amegilla* may play an important role as a pollinator of tropical orchid flora.

### Deceptive pollination and pollinarium reconfiguration in orchids

Both deceptive pollination and pollinarium reconfiguration are considered as effective strategies to promote outcrossing in orchids [2,3,9,13-15], and it appears that selection will become relaxed on one of them if both mechanisms occur in one species due to their antagonistic nature. It seems that this loose selection will lead to two extreme evolutionary outcomes: 1) pollinarium reconfiguration disappears completely in some deceptive orchids with reduced pollinaria (naked or nearly naked pollinarium), such as *Bulbophyllum, Dendrobium*, and *Eria*; and 2) pollinarium reconfiguration is well established in orchids with ample nectar for pollinators, such as *Habenaria* (about approximately 600 species), and *Platanthera* (approximately 150 species). Some orchids, such as *Doritis pulcherrima*, are intermediate between these two extremities. However, we know little about the correlation and evolutionary trends between deceptive pollination and pollinarium reconfiguration.

According to van der Cingel [17,37], Jersakova et al. [13] stated that generalized food-deceptive orchids are reported in 38 genera. Our fieldwork and literature investigation indicated that this number may be underestimated. Many food-deceptive species, such as *AcampeCallostylisCeratostylis, DoritisOxystophyllum, Papilioanthe* and *Sunipia,* are neglected due to the very limited knowledge about their pollination biology. It is likely that generalized food-deceptive orchids occur in approximately 100 genera.

### Breeding strategies of epiphytic orchids in tropical regions

It is well known that the natural fruit sets of food-deceptive orchids are typically pollinator limited [38-40]. Huda & Wilcock [27] indicated that the low fruit set that occurs in tropical epiphytic orchids is associated with the combined effects of a suite of floral and population characteristics, such as rewardless flowers, non-sectile pollinia, self-incompatibility, and small population sizes. Our investigation has indicated that there are at least two mechanisms of promoting fruit set in the food-deceptive epiphytic orchids in tropical regions*:*

(1) Clone-growing life-history. Most members of epiphytic orchids, such as *Bulbophyllum* and *Dendrobium*, are known for their clone-growing life-history (for *D. pulcherrima*, the number of stems per clone was 7.72 ± 9.61 (mean ± SD), n = 101) with several inflorescences flowering synchronously in one clone at anthesis. This can maximize display sizes

(2) Long flowering season. In contrast with temperate orchids, the flowering seasons of many species of epiphytic orchids last from 2 to 3 months (4 months for *D. pulcherrima*), which may result in a high total number of visits, although the visitation rate may be low. As a result, although the natural fruit set is low, the absolute number of fruits may be high for the entire population.

## Competing interest

The authors declare that they have no competing interests.

## Authors’ contributions

Jin X-H and Li D-Z designed the study. Jin X-H and Ren Z-X performed the experiments. Xiang X-G and Jin X-H analyzed the data. Jin X-H, Ren Z-X and Xiang X-G wrote the manuscript, which was further edited by Li D-Z. All authors read and approved the final manuscript.
